# Risk factors of Carbapenem-resistant Enterobacterales intestinal colonization for subsequent infections in hematological patients: a retrospective case-control study

**DOI:** 10.3389/fmicb.2024.1355069

**Published:** 2024-04-12

**Authors:** Zihan Wang, Chunhong Shao, Jing Shao, Yingying Hao, Yan Jin

**Affiliations:** Department of Clinical Laboratory, Shandong Provincial Hospital Affiliated to Shandong First Medical University, Jinan, China

**Keywords:** Carbapenem-resistant Enterobacterales, colonization, infections, risk factors, hematological patients

## Abstract

**Objective:**

Infections caused by Carbapenem-resistant Enterobacterales (CRE) have high treatment costs, high mortality and few effective therapeutic agents. This study aimed to determine the risk factors for progression from intestinal colonization to infection in hematological patients and the risk factors for 30-day mortality in infected patients.

**Methods:**

A retrospective case-control study was conducted in the Department of Hematology at Shandong Provincial Hospital affiliated to Shandong First Medical University from April 2018 to April 2022. Patients who developed subsequent infections were identified as the case group by electronic medical record query of patients with a positive rectal screen for CRE colonization, and patients who did not develop subsequent infections were identified as the control group by stratified random sampling. Univariate analysis and logistic regression analysis determined risk factors for developing CRE infection and risk factors for mortality in CRE-infected patients.

**Results:**

Eleven hematological patients in the study developed subsequent infections. The overall 30-day mortality rate for the 44 hematological patients in the case-control study was 11.4% (5/44). Mortality was higher in the case group than in the control group (36.5 vs. 3.0%, *P* = 0.0026), and septic shock was an independent risk factor for death (*P* = 0.024). Univariate analysis showed that risk factors for developing infections were non-steroidal immunosuppressants, serum albumin levels, and days of hospitalization. In multivariable logistic regression analysis, immunosuppressants [odds ratio (OR), 19.132; 95% confidence interval (CI), 1.349–271.420; *P* = 0.029] and serum albumin levels (OR, 0.817; 95% CI, 0.668–0.999; *P* = 0.049) were independent risk factors for developing infections.

**Conclusion:**

Our findings suggest that septic shock increases mortality in CRE-infected hematological patients. Hematological patients with CRE colonization using immunosuppressive agents and reduced serum albumin are more likely to progress to CRE infection. This study may help clinicians prevent the onset of infection early and take measures to reduce mortality rates.

## 1 Introduction

Carbapenem-resistant Enterobacterales (CRE) infections pose a significant threat to global public health due to their high treatment costs, high mortality, and limited availability of effective therapeutic agents (Xu et al., 2017; European Centre for Disease Prevention and Control, 2018; Tacconelli et al., 2018; Brolund et al., 2019; Centers for Disease Control and Prevention, 2019). A report published in 2023 by the European Center for Disease Prevention and Control (ECDC) and the World Health Organization (WHO) revealed that the prevalence of carbapenem resistance in *Klebsiella pneumoniae* isolates rose by 0, 8, 31, and 20% between 2017 and 2021 (European Centre for Disease Prevention and Control and World Health Organization, 2023). Based on data from the China Antimicrobial Resistance Surveillance System (CARSS), the detection rate of carbapenem-resistant *Klebsiella pneumoniae* (CR-KPN) increased from 10.9% in 2020 to 11.3% in 2021, marking a rise from 6.4% in 2014. Additionally, the national average of *Escherichia coli* resistance to carbapenems remained at 1.6%, the same as in 2020 (China Antimicrobial Resistance Surveillance System, 2023). Patients with hematological disorders are at a higher risk of contracting CRE infections due to their compromised immune systems, low levels of neutrophils, extended hospital stays, undergoing hematopoietic stem cell transplantation, receiving chemotherapy, taking immunosuppressant medications, and frequent use of broad-spectrum antibiotics (Lalaoui et al., 2020; Cao et al., 2021).

As a reservoir for secondary infections, intestinal colonization correlates significantly and independently with CRE infection (Gorrie et al., 2017; Cao et al., 2022). Multiple clinical studies have found that CRE colonization is associated with an increased risk of CRE infection and mortality in patients (Giannella et al., 2014; McConville et al., 2017; Lin et al., 2021; Gomides et al., 2022; Zhu et al., 2022). According to the guidelines from the ECDC, it is advisable to actively screen for CRE and apply effective infection prevention and control strategies to stop the spread of CRE (Magiorakos et al., 2017). Hence, promptly identifying the risk factors contributing to the transition from CRE colonization to subsequent infection will significantly decrease the incidence of CRE infections and mortality.

Fewer studies have been conducted regarding secondary CRE infections in hematological patients with intestinal colonization of CRE. Consequently, this study aimed to identify the factors that increase the likelihood of hematological patients with intestinal CRE colonization progressing to infection, as well as the factors that contribute to mortality in hematological patients already infected with CRE. It provides hematology clinicians with a reference for early identification of high-risk hospitalized patients, allowing them to implement timely preventative measures against CRE infection and mortality.

## 2 Materials and methods

### 2.1 Study design

This retrospective case-control study was conducted at the Shandong Provincial Hospital Affiliated to Shandong First Medical University. This hospital has 3,889 beds, 90 of which belong to the hematology department. This study was approved by the Shandong Provincial Hospital Ethics Committee (NO. SWYX2023-610).

**Figure 1:**
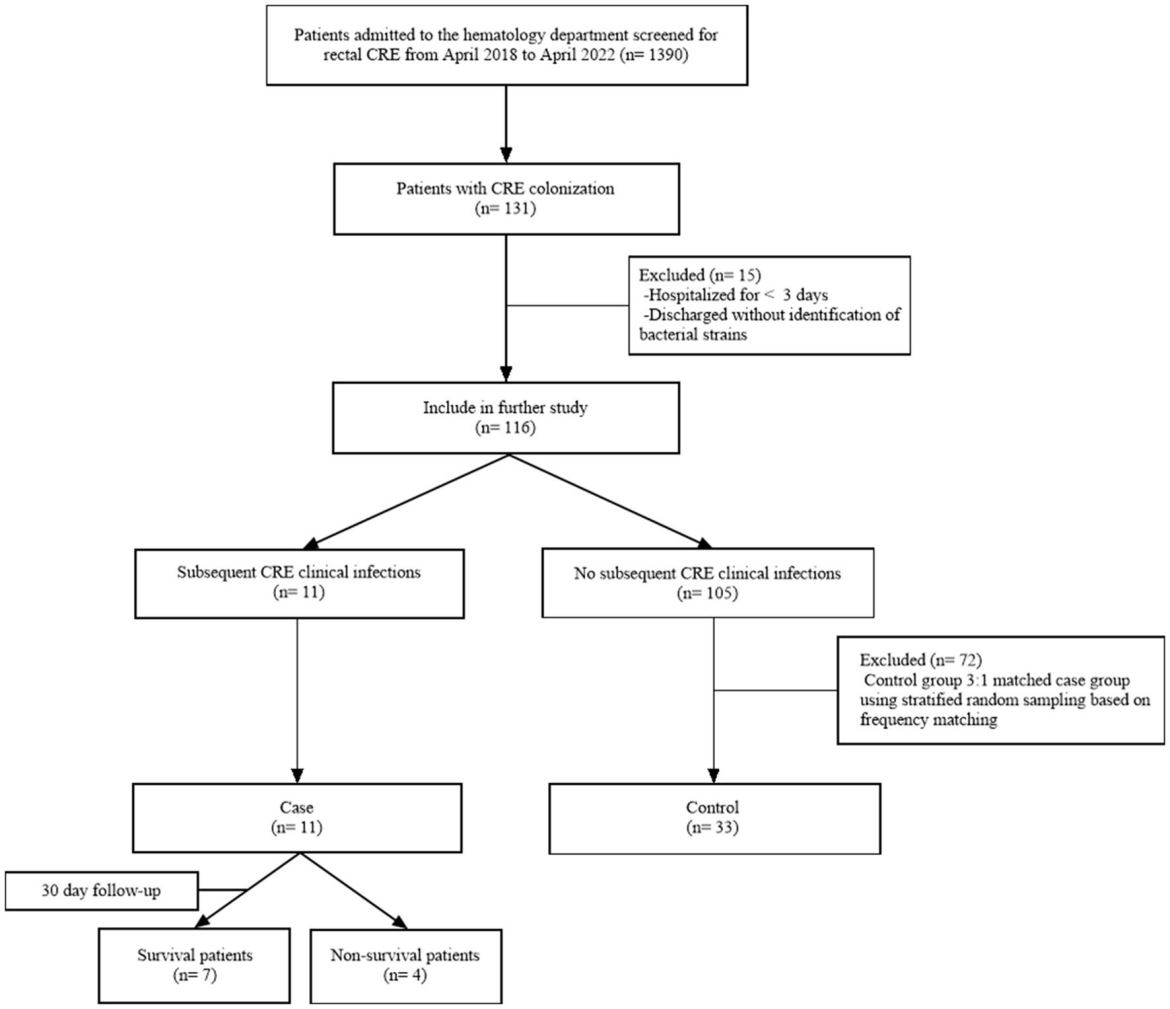
Flowchart of the study design.

CRE rectal colonization was defined as a positive CRE stool screen and the absence of invasive infection. CRE rectal infection was defined as the presence of clinical signs and symptoms of infection and the detection of CRE in specimens taken from the site of infection. Infections such as bacteremia, pneumonia, urinary tract infections, and perianal infections are defined according to guidelines issued by Centers for Disease Control and Prevention (2023). Patients with CRE colonization who were hospitalized for < 3 days and discharged without identifying the colonizing strain were excluded, and patients with positive rectal screening for CRE in the hematology department from April 2018 to April 2022 were further studied. The case group consisted of patients colonized with CRE and subsequently developed CRE infections caused by the same strain (Figure 1). The control group comprised patients selected in a 3:1 ratio relative to the 11 patients in the case group. To enhance the comparability between the case and control groups, we employed frequency matching based on age and sex to mitigate the influence of confounding factors. First, we determined the proportions of age and sex in the case group. Then, we used statistical software to conduct a stratified random sample of colonized patients who did not develop subsequent infections.

The control and case groups were compared using clinical data variables to examine the risk factors associated with subsequent CRE infection. We conducted a 30-day follow-up study on the patients, with death as the end event. The initial event for the control group was the detection of positive test results for CRE colonization samples, while the case group involved detecting positive test results for CRE infection samples. Subsequently, CRE-infected patients were further divided into survival and non-survival groups to analyze risk factors for 30-day mortality. Furthermore, a time-to-event study was conducted to evaluate the characteristics of CRE colonization and infection in CRE-infected patients.

### 2.2 Data collection

Between April 2018 and April 2022, we obtained stool sample data from 1,390 individuals with hematological conditions. Of these, 131 patients had CRE colonization, and we examined their clinical data. Clinical data is obtained from the patient's electronic medical record and encompasses various factors such as demographic characteristics (age and gender), length of hospitalization, hematological diseases (such as acute myeloid leukemia, acute lymphoblastic leukemia, multiple myeloma, myelodysplastic syndrome, lymphoma, etc.), comorbidities (such as diabetes, hypertension, chronic liver disease, gastrointestinal disease, graft vs. host disease, hemorrhagic cystitis, mucositis, pneumonia, diarrhea, and shock), previous invasive procedures (such as deep venous catheterization, urinary catheterization, hematopoietic stem cell transplantation), exposure to drugs (such as chemotherapy, glucocorticoids, non-steroidal immunosuppressants, proton pump inhibitors, carbapenems, cephalosporins, fluoroquinolones, aminoglycosides, glycopeptide, penicillins, tigecycline), laboratory examinations (neutrophil count and serum albumin levels), CRE isolates (such as *Escherichia coli, Klebsiella pneumoniae, Enterobacter cloacae*, and others), and outcomes (mortality at 30 days). Furthermore, we collected *in vitro* susceptibility data, carbapenemase phenotypes (serine carbapenemase and metal β-lactamases), sensitive antibiotic treatments, and the timing of colonization and infection in patients infected with CRE.

### 2.3 Microbiology

Bacterial isolates for this study were identified using the Vitek 2 automatic system (bioMérieux, France). The stool samples were inoculated on MacConkey agar plates (ThermoFisher, USA), followed by carbapenem antimicrobial susceptibility testing using the disk diffusion method to confirm the presence of carbapenem-resistant Enterobacteriaceae (CRE). CRE refers to Enterobacterales that exhibit resistance to at least one carbapenem antibiotic, namely ertapenem, imipenem, or meropenem. The broth microdilution method or the Vitek 2 system determined antimicrobial susceptibility tests. Tigecycline susceptibility was determined using the US Food and Drugs Administration (FDA) interpretive criteria (US Food Drug and Administration, 2023). Colistin using the European Committee on Antimicrobial Susceptibility Testing (EUCAST) breakpoint (European Committee on Antimicrobial Susceptibility Testing, 2023), and the remaining susceptibility results were interpreted using the Clinical and Laboratory Standards Institute (CLSI) documentation standards (Clinical and Laboratory Standards Institute, 2022). The Modified Carbapenem Inactivation Method (mCIM) and Modified EDTA-Carbapenem Inactivation Method (eCIM) were employed to detect carbapenemase phenotypes (Pierce et al., 2017).

### 2.4 Statistical analysis

All statistical analyses were performed using IBM SPSS Statistics version 25.0 software. We first applied the Shapiro-Wilk test for normality to the continuous variables. Continuous variables that were normally distributed with variance equivalence were denoted as mean ± standard deviation (SD) and analyzed using independent samples *t*-test. While continuous variables that were not normally distributed or had non-equivalent variances were denoted as median with interquartile ranges (IQR) and analyzed using the Mann-Whitney *U*-Test. Categorical variables were analyzed using the Pearson Chi-square test, Continuity correction test, or Fisher's Exact Test. Variables with *P* < 0.05 in the univariate analysis were included in the logistic regression analysis after Collinearity diagnostics and Variance inflation factor (VIF) checks to exclude multicollinearity. Kaplan-Meier curves and the log-rank test for the CRE colonization group vs. the infection group performed survival analysis. All tests were two-tailed and *P* < 0.05 indicated statistical significance.

## 3 Results

### 3.1 Patient characteristics

**Figure 2:**
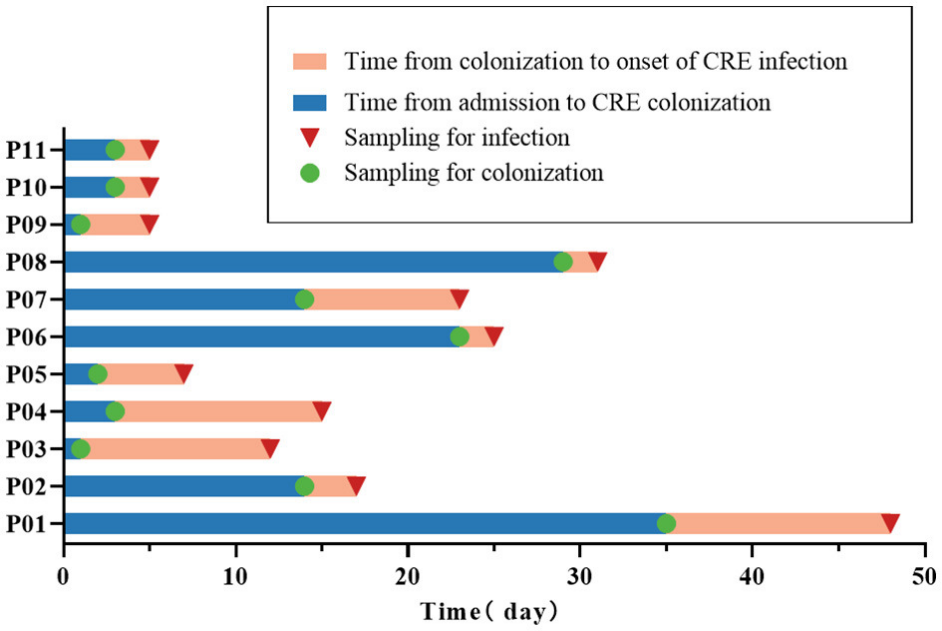
Time characteristics of colonization and infection in 11 CRE-infected patients. The 11 CRE-infected patients are denoted as P01-P11.

A total of 131 patients were identified as positive for CRE colonization in this study (131/1,390, 9.4%), and the colonization frequency has been seen to increase annually (Supplementary Figure 1). After applying the exclusion criteria, 116 patients with CRE colonization were selected for further study. Eleven patients with CRE colonization subsequently acquired CRE infection (11/116, 9.5%). A control group of 33 out of the 105 patients colonized with CRE but did not acquire subsequent infection was selected using stratified random sampling. This control group was included in the case-control research alongside the group of infected patients (Figure 1). There was a higher proportion of males than females in the case and control groups (both 63.6%). The age of the case group was 35.36 ± 13.44 years, and the age of the control group was 36.33 ± 12.78 years. Acute myeloid leukemia was the most prevalent hematological condition in the case group (5/11, 45.5%), with acute lymphoblastic leukemia being the second most common (4/11, 36.4%). The control group primarily comprised individuals with acute myeloid leukemia (15/33, 45.5%), lymphoma (9/33, 27.3%), and acute lymphoblastic leukemia (3/33, 9.1%). Furthermore, we analyzed the time characteristics of 11 CRE-infected patients from admission to colonization and subsequent infection. The median duration from admission to colonization was 3 days (2–23 days). Similarly, the median duration from colonization to infection was 4 days (2–11 days; Figure 2).

### 3.2 Microbiological characteristics

**Figure 3:**
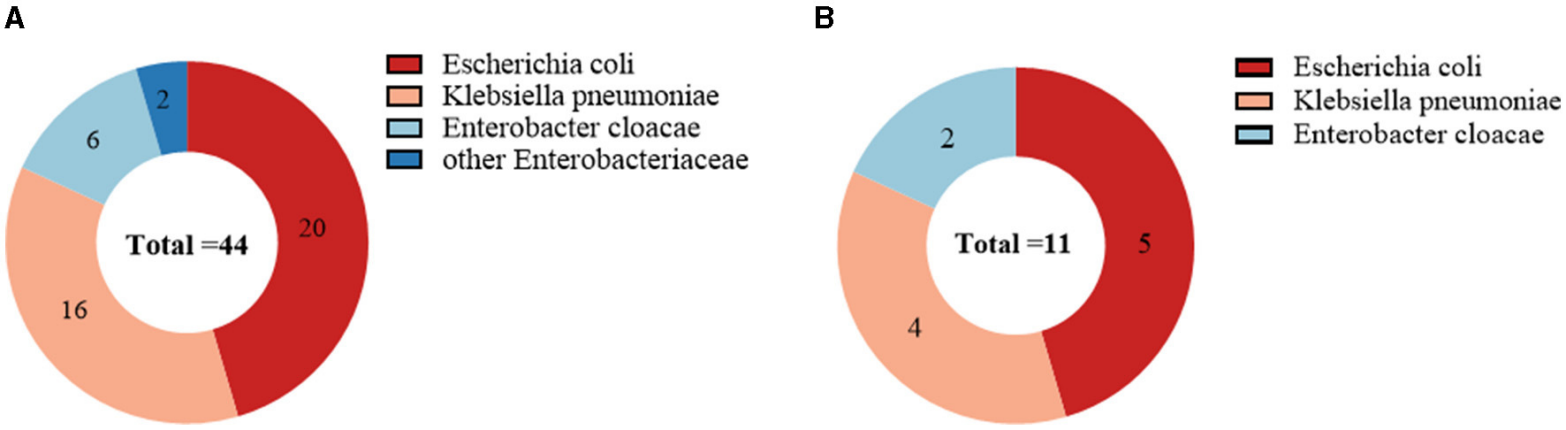
Microbial characterization of CRE strains isolated from 44 CRE-colonized patients **(A)**; Microbial characterization of CRE strains isolated from 11 CRE-infected patients **(B)**. *Escherichia coli* was the most common strain for CRE colonization and infection, followed by *Klebsiella pneumoniae*.

**Figure 4:**
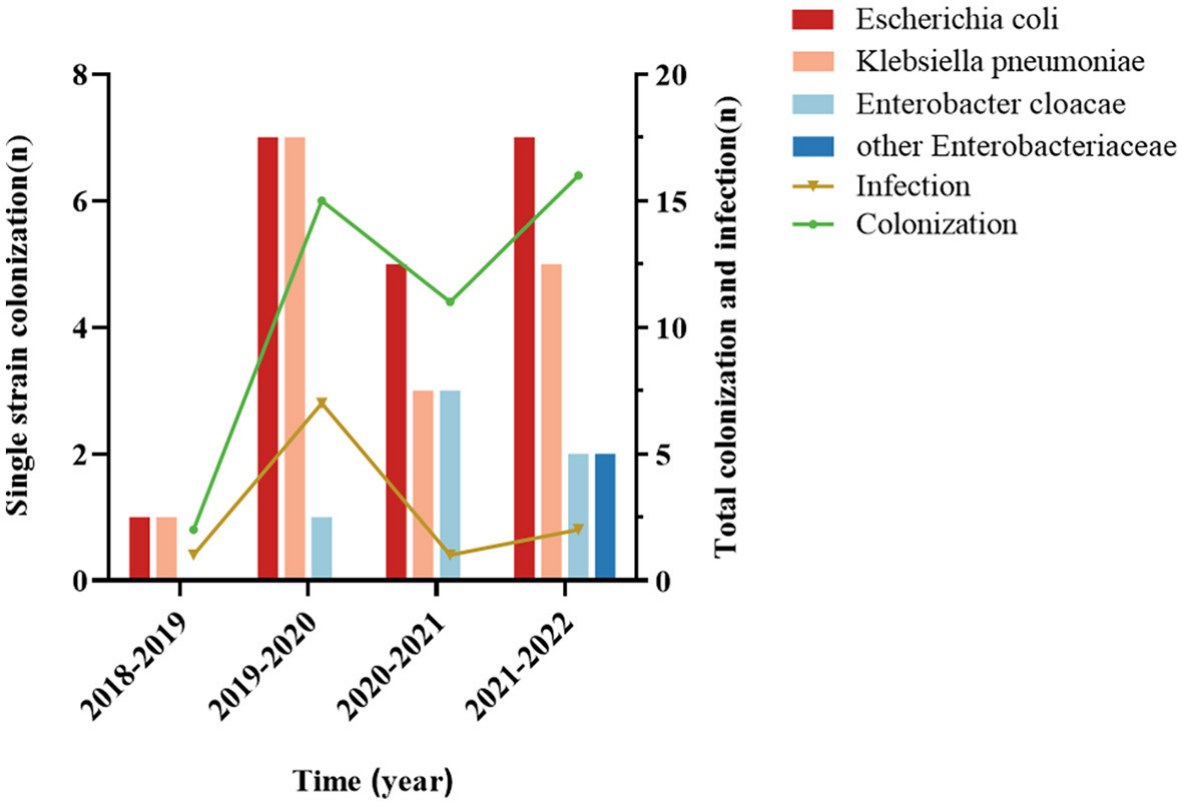
Trends of CRE colonization and infection in case-control studies, annual distribution of CRE-colonized strains. The annual trend of CRE colonization was upward, and the *Escherichia coli* counts were significantly different from the other strains.

Of the 44 CRE-colonized patients, 20 were colonized with *Escherichia coli* (20/44, 45.5%), 16 with *Klebsiella pneumoniae* (16/44, 36.4%), 6 with *Enterobacter cloacae* (6/44, 13.6%), and two with others (2/44, 4.5%). *Escherichia coli* was the most prevalent among patients with secondary CRE infections (5/11, 45.5%), followed by *Klebsiella pneumoniae* (4/11, 36.4%; Figure 3). Out of the 11 cases, 72.7% (8/11) were caused by infectious strains originating from bacteremia, while perianal infections, pneumonia, and urinary tract infections each accounted for 9% (1/11). In addition, we found that CRE colonization showed an overall increasing trend from year to year. *Escherichia coli* was the dominant strain among the colonizers in the hematology department each year (Figure 4).

Based on the CLSI and FDA breakpoints, the infected group showed the greatest susceptibility to colistin (100%) among CRE, followed by tigecycline and amikacin (both 81.8%) and gentamicin (63.6%). 72.7% of CRE exhibited resistance to aztreonam, while 72.7% of CRE showed resistance or intermediate susceptibility to tobramycin. All CRE found in infected patients exhibited resistance to a wide range of antibiotics, including ampicillin, ampicillin-sulbactam, piperacillin-tazobactam, cefoperazone-sulbactam, cefazolin, cefuroxime, ceftazidime, ceftriaxone, cefepime, cefoxitin, cefotetan, ertapenem, imipenem, meropenem, ciprofloxacin, levofloxacin, and trimethoprim/sulfamethoxazole (Supplementary Table 1). In addition, the carbapenemase phenotypes of CRE-infected patients were characterized. Of these, seven strains produced metal β-lactamases (7/11, 63.6%), and four strains produced serine carbapenemases (4/11, 36.4%; Supplementary Table 2).

### 3.3 Analysis of risk factors for progression to CRE infection

Table 1 displays the findings of the risk factor analysis for the progression from CRE colonization to CRE infection. No significant differences were observed between the case and control groups regarding the prevalence of hematological conditions, comorbidities, invasive operations, and CRE strains (all *P* > 0.05). The median duration of hospitalization in the case group was 29 days (21–47 days), significantly longer than the control group's median duration of 11 days (6–26 days). Additionally, the mean albumin level in the case group was 28.11 ± 3.61 g/L, lower than the mean albumin level of 34.64 ± 6.92 g/L in the control group. In univariate analyses, variables associated with progression to infection included length of hospitalization, application of non-steroidal immunosuppressants, and serum albumin level. In multivariate logistic regression analysis, immunosuppressants (OR, 19.132; 95% CI, 1.349–271.420; *P* = 0.029) and albumin level (OR, 0.817; 95% CI, 0.668–0.999; *P* = 0.049) were identified as independent risk factors.

**Table 1:** Univariate analysis and multivariate logistic regression analysis of risk factors for progression of CRE colonization to CRE infection.

**Characteristics**	**Univariable analysis**			**Multivariate analysis**	
	**Case**	**Control**	***P*** **-value**	**OR (95% CI)**	***P*** **-value**
	***n*** = **11 (%)**	***n*** = **33 (%)**			
**Sex-male**	7 (63.6)	21 (63.6)			
**Age (years), mean** **±SD**	35.36 ± 13.44	36.33 ± 12.78			
**Hospital stay (days), median (IQR)**	29 (21.47)	11 (6.26)	0.004^*^	1.062 (0.996–1.132)	0.066
**Hematological disease**					
Acute myeloid leukemia	5 (45.5)	15 (45.5)	1.000		
Acute lymphoblastic leukemia	4 (36.4)	3 (9.1)	0.096		
Lymphoma	0 (0.0)	9 (27.3)	0.131		
Myelodysplastic syndrome	1 (9.1)	2 (6.1)	1.000		
Multiple myeloma	0 (0.0)	2 (6.1)	1.000		
Others	1 (9.1)	2 (6.1)	1.000		
**Comorbidities**					
Diabetes	1 (9.1)	2 (6.1)	1.000		
Hypertension	0 (0.0)	3 (9.1)	0.561		
Chronic liver disease	0 (0.0)	4 (12.1)	0.545		
Gastrointestinal disease	3 (27.3)	5 (15.2)	0.652		
GVHD	2 (18.2)	3 (9.1)	0.784		
Hemorrhagic cystitis	1 (9.1)	3 (9.1)	1.000		
Mucositis	7 (63.6)	10 (30.3)	0.108		
Pneumonia	9 (81.8)	25 (75.8)	1.000		
Diarrhea	5 (45.5)	6 (18.2)	0.159		
Shock	0 (0.0)	1 (3.0)	1.000		
**Previous invasive procedures**					
Deep venous catheterization	7 (63.6)	10 (30.3)	0.108		
Urinary catheterization	1 (9.1)	1 (3.0)	0.442		
HSCT	3 (27.3)	3 (9.1)	0.310		
**Exposure to drug**					
Carbapenems ( ≤ 90 days)	10 (90.9)	23 (69.7)	0.315		
Cephalosporins ( ≤ 90 days)	8 (72.7)	19 (57.6)	0.592		
Fluoroquinolones ( ≤ 90 days)	3 (27.3)	13 (39.4)	0.717		
Aminoglycosides ( ≤ 90 days)	2 (18.2)	9 (27.3)	0.841		
Glycopeptides ( ≤ 90 days)	2 (18.2)	10 (30.3)	0.696		
Penicillins ( ≤ 90 days)	2 (18.2)	10 (30.3)	0.696		
Tigecycline ( ≤ 90 days)	5 (45.5)	9 (27.3)	0.455		
Chemotherapy ( ≤ 30 days)	9 (81.8)	22 (66.7)	0.567		
Glucocorticoids ( ≤ 30 days)	9 (81.8)	19 (57.6)	0.278		
Non-steroidal immunosuppressants ( ≤ 30 days)	10 (90.9)	12 (36.4)	0.002^*^	19.132 (1.349–271.420)	0.029^*^
PPIs ( ≤ 30 days)	6 (54.5)	21 (63.6)	0.858		
**Laboratory examinations**					
Neutrophils ( × 10^9^/L), median (IQR)	0.56 (0.03, 3.03)	2.41 (0.93, 3.94)	0.080		
Albumin (g/L), mean ± SD	28.11 ± 3.61	34.64 ± 6.92	0.005^*^	0.817 (0.668–0.999)	0.049^*^
**CRE isolates**					
*Escherichia coli*	5 (45.5)	15 (45.5)	1.000		
*Klebsiella pneumoniae*	4 (36.4)	12 (36.4)	1.000		
*Enterobacter cloacae*	2 (18.2)	4 (12.1)	1.000		
Other Enterobacterales	0 (0.0)	2 (6.1)	1.000		

^*^Significant statistical difference (P < 0.05).

P, test significance; OR, odds ratio; CI, confidence interval; SD, standard deviation; IQR, Interquartile range; GVHD, graft vs. host disease; HSCT, hematopoietic stem cell transplantation; PPIS, proton pump inhibitors.

### 3.4 Analysis of risk factors for 30-day mortality in CRE-infected patients

**Figure 5:**
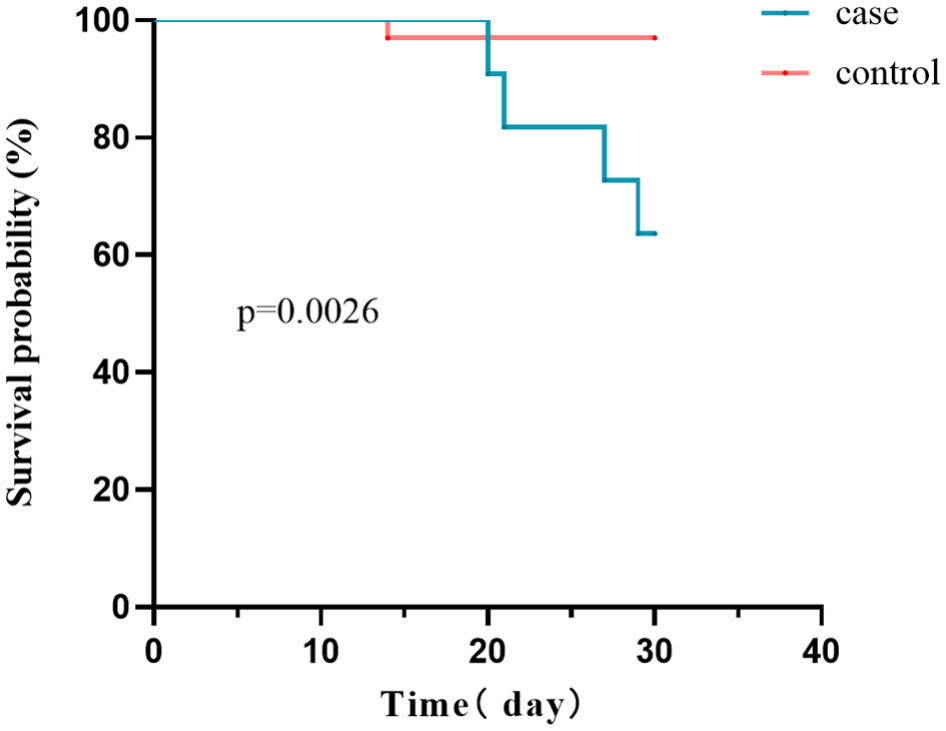
Survival curves of the case and control groups. The 30-day mortality rate was considerably higher in CRE-infected patients than in CRE-colonized patients (36.5 vs. 3.0%, *P* = 0.0026).

A total of five patients in both the case and control groups died during the 30-day follow-up period (5/44, 11.4%). The case group's mortality rate was significantly higher than the control group (36.5 vs. 3.0%, *P* = 0.0026; Figure 5). Within 30 days, four patients infected with CRE died of bacteremia. In order to determine mortality risk factors among hematological patients infected with CRE, we conducted a comparative analysis of the survival and non-survival groups using the same variables outlined in Table 1. Furthermore, we considered combined carbapenems, *in vitro* sensitive antibiotic treatment, and carbapenemase phenotypes (Supplementary Table 2). Four patients were treated with monotherapy (tigecycline, polymyxin, or aminoglycoside), whereas seven other patients were treated with combination therapy (all combined with tigecycline). Carbapenems were administered in combination to nine patients. Univariate analysis revealed that patients with CRE infection complicated by septic shock had a higher likelihood of mortality. Furthermore, no statistically significant distinction was observed between monotherapy and combination therapy.

## 4 Discussion

Controlling the spread of CRE has become a critical public health concern on a global scale, necessitating the implementation of infection prevention and control measures (Magiorakos et al., 2017; Zeng et al., 2023). Intensive Care Unit (ICU) admission and hematological malignancies are identified as risk factors associated with CRE infection (Tian et al., 2016; Chen et al., 2022; Zhang et al., 2022). Multiple studies have investigated the factors that increase the risk of infection and mortality from CRE in patients with either ICU or hematological malignancies (McConville et al., 2017; Kontopoulou et al., 2019; Lin et al., 2021; Chen et al., 2023). The hematology department admits many immunocompromised patients with hematological disorders who are more vulnerable to CRE infections. Our study's infection rate in patients with CRE colonization was 9.5% (11/116). In patients colonized with CRE, the prevalence of CRE infection varied between 9.1 and 86% (Borer et al., 2012; Schechner et al., 2013; Dickstein et al., 2016; Tischendorf et al., 2016; Gomides et al., 2022). Due to the different types of diseases and treatments administered to patients in each department, the results may be inconsistent; therefore, it is essential to examine hematology departments separately for CRE infection risk factors. Additionally, CRE colonization is a critical factor in CRE infection, which does not occur in all patients with colonization (Gorrie et al., 2017; Qin et al., 2020). Only a limited number of studies have been conducted concerning the risk factors that contribute to CRE infection in patients with hematological diseases (Zhang et al., 2019). Our study aims to identify the risk variables associated with the progression from colonization to infection in hematological patients and the risk factors for mortality in infected patients. These could provide clinicians with recommendations for preventing and controlling CRE infections.

The two studies on hematological malignancies had CRE colonization rates of 6.56 and 10.3%, respectively (Zhu et al., 2022; Chen et al., 2023). The CRE colonization rate in studies conducted in the ICU ranged from 15.5 to 45.4% (McConville et al., 2017; Kontopoulou et al., 2019; Gomides et al., 2022). The CRE colonization rate in this study was 9.4% (131/1390), similar to the studies on hematological malignancies and lower than the ICU colonization rate. A prospective study also suggests intestinal CRE colonization is more prevalent in the ICU, with widespread rapid spread (Chu et al., 2022). Furthermore, Cao et al. (2022) observed that the CRE colonization rate among recipients of allogeneic hematopoietic stem cell transplantation would be marginally elevated at 23.8%. The colonization rate exhibits variation within different departments and may be associated with pharmacological therapies, patient groups, and clinical settings.

Our study also observed an overall trend of increasing CRE colonization in hematological patients throughout the years, aligning with the findings of a multicenter investigation (Fasciana et al., 2023). Nevertheless, the growth rate in 2020–2022 is considerably diminished compared to 2018–2020. This can be ascribed to the proactive implementation of the national policy regarding the rational utilization of antimicrobial drugs in clinical practice and the enhanced surveillance of hospital infections by medical institutions in recent years (China Antimicrobial Resistance Surveillance System, 2023). Medical institutions must prioritize improving the appropriate utilization of antimicrobial drugs, minimizing the excessive use of broad-spectrum antimicrobial medicines like carbapenems, and effectively implementing hospital infection control measures to contain the widespread transmission of drug-resistant bacteria.

Wang et al.'s (2018) longitudinal large-scale CRE data revealed that the predominant strain of clinical CRE isolates was *Klebsiella pneumoniae*, which exhibited a yearly upward trend. *Escherichia coli* was the primary strain that colonized the hematology department of our hospital, both in terms of total colonization and colonization on an annual basis. The major strain of secondary CRE infection was *Escherichia coli*, followed by *Klebsiella pneumoniae*, consistent with a prior study conducted in the Department of Hematology (Zhang et al., 2019). The infected strains mainly produced metal β-lactamases, and the choice of antibiotics varied for different carbapenemases (Wang et al., 2018). KPC is a serine enzyme that hydrolyzes aztreonam but can be inhibited by the novel enzyme inhibitors avibactam and vebobactam, whereas NDM is a metalloenzyme that does not hydrolyze aztreonam but is not inhibited by avibactam and vebobactam. Hence, it is critical to identify the carbapenemase phenotype for subsequent antimicrobial treatment.

Chemotherapy, invasive operations, ICU admission, prolonged hospitalization, and exposure to carbapenem antibiotics are common risk factors for acquiring infections in patients with CRE colonization (Schechner et al., 2013; McConville et al., 2017; Collingwood et al., 2020; Chu et al., 2022; Chen et al., 2023). In our univariate analysis, prolonged hospitalization was identified as a risk factor for developing infections in patients harboring CRE colonization. Nevertheless, when doing multivariate analyses, the duration of hospitalization was not shown to be statistically significant. Instead, non-steroidal immunosuppressants and albumin levels were identified as independent risk factors for the progression of infections.

While prior use of carbapenems was a common culprit, and chemotherapy as well as proton pump inhibitors (PPIs) have also been found to be associated with infections (Chen et al., 2023), our analysis of previous drug exposures showed that immunosuppressant use significantly increased the risk of infection in patients (OR, 19.132; 95% CI, 1.349–271.420; *p* = 0.029). Moreover, a recent international matched case-control-control study found that immunosuppressive drugs in an inpatient population were risk factors for CRE infection (Perez-Galera et al., 2023). Many individuals with weakened immune systems due to hematological malignancies and hematopoietic stem cell transplants are admitted to hematology departments. These patients are regularly exposed to chemotherapy and immunosuppressive medications (Bar-Yoseph et al., 2019). Myelosuppression, an adverse effect, can be experienced with any immunosuppressive drug and may result in agranulocytosis, hence increasing vulnerability to infections (Fraiser et al., 1991; Lee et al., 2016; Toksvang et al., 2022). Furthermore, reducing albumin levels amplifies the susceptibility to infection in patients with CRE colonization (Rao et al., 2020; Liu et al., 2022; Qian et al., 2023). Critically sick patients often exhibit hypoalbuminemia, which is characterized by a gradual depletion of vital protein components in the body due to inflammation caused by infection (McMillan et al., 2001). Hence, it is imperative to exercise caution in administering immunosuppressive medicines and promptly address hypoalbuminemia in patients with CRE colonization to decrease CRE infections effectively.

In the present study, the 30-day mortality rate of patients with CRE infection was 36.4%, similar to that in studies on patients with hematological diseases (Liu et al., 2019; Zhang et al., 2019; Chen et al., 2023). The independent risk factor for death was septic shock, a variable that has been found to be associated with high mortality in several previously conducted studies (Tumbarello et al., 2012; Daikos et al., 2014; Chen et al., 2022, 2023). Septic shock is a sign of severe infection, and critically ill patients are more likely to die after the onset of the disease. Our study found that combination therapy did not reduce patient mortality, which is inconsistent with previous studies (Daikos et al., 2014; Tumbarello et al., 2015; Trecarichi et al., 2016; Chen et al., 2022). However, Paul et al. propose that patients infected with pathogens that exhibit limited susceptibility to antibiotics *in vitro* are more likely to be prescribed monotherapy. Conversely, patients infected with microorganisms more susceptible to *in vitro* susceptibility antibiotics are more likely to receive combination therapy. Patients with multiple antibiotic resistance may be more severely ill at baseline than patients with less antibiotic resistance. Consequently, the comparisons made between monotherapy and combination therapy may be influenced by selection bias (Paul et al., 2014). Our findings indicate that individuals with clinically severe disease had a higher likelihood of being prescribed combination therapies. Furthermore, the sample size of patients receiving combination therapy is small. Therefore, the effect of combination therapy needs to be evaluated in randomized controlled trials.

This study has some limitations. First, the study was single-center, and the results may not be generalizable to other departments and regions. Second, the study was retrospective and could not determine that the deaths were caused entirely by the CRE, thus making it impossible to analyze the attributable mortality rates. Third, the small sample size of infection and bias in treatment selection had limited ability to analyze risk factors for death. Therefore, multicenter prospective studies are necessary to address these issues.

## 5 Conclusion

In summary, the results of our study suggest that careful use of non-steroidal immunosuppressive agents and prompt correction of reduced albumin levels in hematological patients with CRE colonization can help reduce the incidence of CRE infections. Septic shock leads to a significant increase in mortality in patients with CRE infection. These findings may help clinicians take appropriate precautions to reduce the incidence of CRE infections and decrease mortality in such patients.

## Data availability statement

The original contributions presented in the study are included in the article/Supplementary material, further inquiries can be directed to the corresponding author.

## Ethics statement

The studies involving humans were approved by Biomedical Research Ethic Committee of Shandong Provincial Hospital. The studies were conducted in accordance with the local legislation and institutional requirements. The human samples used in this study were acquired from primarily isolated as part of your previous study for which ethical approval was obtained. Written informed consent for participation was not required from the participants or the participants' legal guardians/next of kin in accordance with the national legislation and institutional requirements.

## Author contributions

ZW: Writing – original draft, Software, Methodology, Investigation, Formal analysis, Data curation. CS: Writing – review & editing, Supervision, Resources. JS: Writing – review & editing, Supervision, Resources. YH: Writing – review & editing, Supervision, Resources. YJ: Resources, Writing – review & editing, Supervision, Funding acquisition, Conceptualization.
