# Supplementary material for: Risk factors of Carbapenem-resistant Enterobacterales intestinal colonization for subsequent infections in hematological patients: a retrospective case-control study
Source: Front Microbiol. 2024 Apr 12;15:1355069. doi: 10.3389/fmicb.2024.1355069 (PMC11045900; doi:10.3389/fmicb.2024.1355069)
Supplement: Supplementary file 1 [file Data_Sheet_1.docx]

Supplementary Material

**
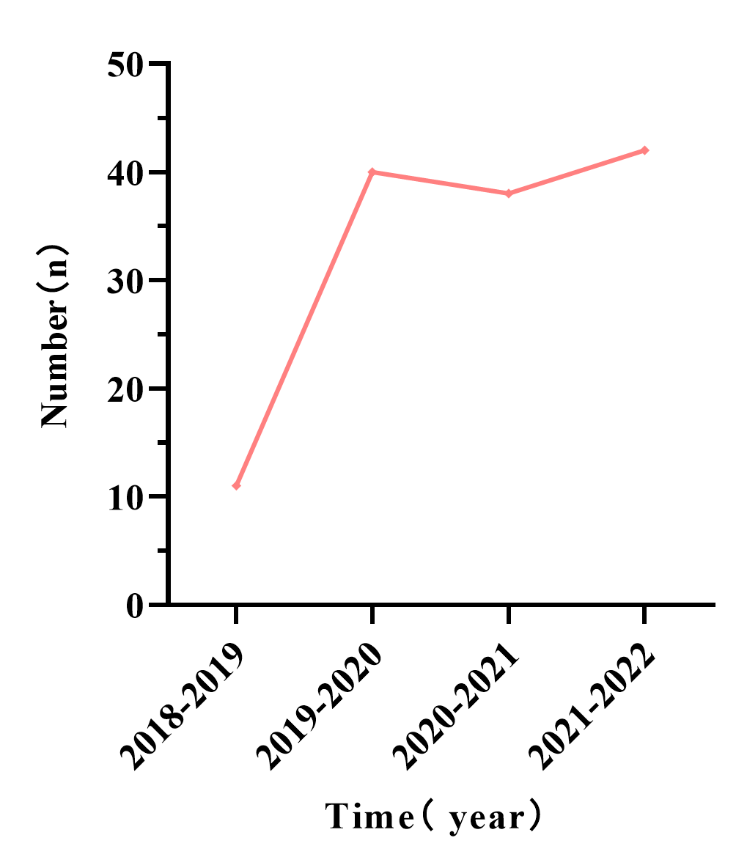
**

**Supplementary Figure 1.** Yearly trend in total CRE colonization. The growth rate in 2020-2022 is significantly lower than in 2018-2020.

**Supplementary Table 1** In vitro susceptibility results of 11 patients with CRE infection

| **Antibiotic types** | **Resistance**  **[n (%)]** | **Intermediate**  **[n (%)]** | **Susceptibility**  **[n (%)]** |
| --- | --- | --- | --- |
| Ampicillin | 11(100.0) | 0(0.0) | 0(0.0) |
| Ampicillin-sulbactam | 11(100.0) | 0(0.0) | 0(0.0) |
| Piperacillin-tazobactam | 9(81.8) | 2(18.2) | 0(0.0) |
| Cefoperazone-sulbactam | 11(100.0) | 0(0.0) | 0(0.0) |
| Cefazolin | 11(100.0) | 0(0.0) | 0(0.0) |
| Cefuroxime | 11(100.0) | 0(0.0) | 0(0.0) |
| Ceftazidime | 11(100.0) | 0(0.0) | 0(0.0) |
| Ceftriaxone | 11(100.0) | 0(0.0) | 0(0.0) |
| Cefepime | 11(100.0) | 0(0.0) | 0(0.0) |
| Cefoxitin | 11(100.0) | 0(0.0) | 0(0.0) |
| Cefotetan | 11(100.0) | 0(0.0) | 0(0.0) |
| Aztreonam | 8(72.7) | 0(0.0) | 3(27.3) |
| Ertapenem | 11(100.0) | 0(0.0) | 0(0.0) |
| Imipenem | 11(100.0) | 0(0.0) | 0(0.0) |
| Meropenem | 11(100.0) | 0(0.0) | 0(0.0) |
| Amikacin | 2(18.2) | 0(0.0) | 9(81.8) |
| Gentamicin | 4(36.4) | 0(0.0) | 7(63.6) |
| Tobramycin | 3(27.3) | 5(45.4) | 3(27.3) |
| Ciprofloxacin | 11(100.0) | 0(0.0) | 0(0.0) |
| Levofloxacin | 9(81.8) | 2(18.2) | 0(0.0) |
| Trimethoprim/sulfamethoxazole | 11(100.0) | 0(0.0) | 0(0.0) |
| Tigecycline | 2(18.2) | 0(0.0) | 9(81.8) |
| Colistin | 0(0.0) | 0(0.0) | 11(100.0) |

**Supplementary Table 2** Risk factors for 30-day mortality in CRE-infected patients

| **Characteristics** | **Survival**  **(n= 7)** | **Non-survival**  **(n= 4)** | **P-value** |
| --- | --- | --- | --- |
| **Sex-Male** | 5(71.4) | 2(50.0) | 0.576 |
| **Age (years), mean ± SD** | 39.29±13.43 | 28.5±11.96 | 0.217 |
| **Hospital stay (days),**  **mean ± SD** | 38±20.75 | 29.5±9.434 | 0.466 |
| **Hematological disease** | | | |
| Acute myeloid leukemia | 3(42.9) | 2(50.0) | 1.000 |
| Acute lymphoblastic leukemia | 3(42.9) | 1(25.0) | 1.000 |
| Myelodysplastic syndrome | 1(14.3) | 0(0.0) | 1.000 |
| Others | 0(0.0) | 1(25.0) | 0.364 |
| **Comorbidities** | | | |
| Diabetes | 1(14.3) | 0(0.0) | 1.000 |
| Gastrointestinal disease | 1(14.3) | 2(50.0) | 0.491 |
| GVHD | 2(28.6) | 0(0.0) | 0.491 |
| Hemorrhagic cystitis | 1(14.3) | 0(0.0) | 1.000 |
| Mucositis | 4(57.1) | 3(75.0) | 1.000 |
| Pneumonia | 6(85.7) | 3(75.0) | 1.000 |
| Diarrhea | 3(42.9) | 2(50.0) | 1.000 |
| Septic shock | 0(0.0) | 3(75.0) | 0.024* |
| **Previous invasive procedures** | | | |
| Deep venous catheterization | 4(57.1) | 3(75.0) | 1.000 |
| Urinary catheterization | 1(14.3) | 0(0.0) | 1.000 |
| HSCT | 2(28.6) | 1(25.0) | 1.000 |
| **Exposure to drug** | | | |
| Carbapenems (≤90 days) | 6(85.7) | 4(100.0) | 1.000 |
| Cephalosporins (≤90 days) | 4(57.1) | 4(100.0) | 0.236 |
| Fluoroquinolones (≤90 days) | 1(14.3) | 2(50.0) | 0.491 |
| Aminoglycosides (≤90 days) | 0(0.0) | 2(50.0) | 0.109 |
| Glycopeptides (≤90 days) | 0(0.0) | 2(50.0) | 0.109 |
| Penicillins (≤90 days) | 1(14.3) | 1(25.0) | 1.000 |
| Tigecycline (≤90 days) | 3(42.9) | 2(50.0) | 1.000 |
| Chemotherapy (≤30 days) | 5(71.4) | 4(100.0) | 0.491 |
| Glucocorticoids (≤30 days) | 6(85.7) | 3(75.0) | 1.000 |
| non-steroidal immunosuppressants  (≤30 days) | 7(100.0) | 3(75.0) | 0.364 |
| PPIs (≤30 days) | 4(57.1) | 2(50.0) | 1.000 |
| **Sensitive antibiotics treatment** | | | |
| Tigecycline +aminoglycoside + polymyxin | 1(14.3) | 2(50.0) | 0.491 |
| Tigecycline + aminoglycoside | 3(42.9) | 0(0.0) | 0.236 |
| Tigecycline + polymyxin | 0(0.0) | 1(25.0) | 0.364 |
| Tigecycline | 1(14.3) | 0(0.0) | 1.000 |

**Supplementary Table 2 (continued)**

| **Characteristics** | **Survival**  **(n= 7)** | **Non-survival**  **(n= 4)** | **P-value** |
| --- | --- | --- | --- |
| Polymyxin | 1(14.3) | 1(25.0) | 1.000 |
| Aminoglycoside | 1(14.3) | 0(0.0) | 1.000 |
| **Combined carbapenems** | 5(71.4) | 4(100.0) | 0.491 |
| **Laboratory examinations** | | | |
| Neutrophils(×10^9^/L), median (IQR) | 2.75(0.04,3.03) | 0.135(0.03,0.48) | 0.164 |
| Albumin (g/L), mean ± SD | 26.96±3.82 | 30.13±2.38 | 0.172 |
| **CRE isolates** | | | |
| Escherichia coli | 2(28.6) | 2(50.0) | 0.576 |
| Klebsiella pneumoniae | 3(42.9) | 2(50.0) | 1.000 |
| Enterobacter cloacae | 2(28.6) | 0(0.0) | 0.491 |
| **Carbapenemase phenotypes** | | | |
| Serine carbapenemase | 2(28.6) | 2(50.0) | 0.576 |
| Metal β-lactamase | 5(71.4) | 2(50.0) | 0.576 |
